# A Mathematical Framework for Estimating Pathogen Transmission Fitness and Inoculum Size Using Data from a Competitive Mixtures Animal Model

**DOI:** 10.1371/journal.pcbi.1002026

**Published:** 2011-04-28

**Authors:** James M. McCaw, Nimalan Arinaminpathy, Aeron C. Hurt, Jodie McVernon, Angela R. McLean

**Affiliations:** 1 Vaccine and Immunisation Research Group, Murdoch Childrens Research Institute, Royal Children's Hospital, Parkville, Victoria, Australia; 2 Melbourne School of Population Health, The University of Melbourne, Victoria, Australia; 3 Department of Ecology and Evolutionary Ecology, Princeton University, New Jersey, United States of America; 4 WHO Collaborating Centre for Reference and Research on Influenza, North Melbourne, Victoria, Australia; 5 Institute for Emerging Infections, Department of Zoology, University of Oxford, Oxford Martin School, Oxford, United Kingdom; Imperial College London, United Kingdom

## Abstract

We present a method to measure the relative transmissibility (“transmission fitness”) of one strain of a pathogen compared to another. The model is applied to data from “competitive mixtures” experiments in which animals are co-infected with a mixture of two strains. We observe the mixture in each animal over time and over multiple generations of transmission. We use data from influenza experiments in ferrets to demonstrate the approach. Assessment of the relative transmissibility between two strains of influenza is important in at least three contexts: 1) Within the human population antigenically novel strains of influenza arise and compete for susceptible hosts. 2) During a pandemic event, a novel sub-type of influenza competes with the existing seasonal strain(s). The unfolding epidemiological dynamics are dependent upon both the population's susceptibility profile and the inherent transmissibility of the novel strain compared to the existing strain(s). 3) Neuraminidase inhibitors (NAIs), while providing significant potential to reduce transmission of influenza, exert selective pressure on the virus and so promote the emergence of drug-resistant strains. Any adverse outcome due to selection and subsequent spread of an NAI-resistant strain is exquisitely dependent upon the transmission fitness of that strain. Measurement of the transmission fitness of two competing strains of influenza is thus of critical importance in determining the likely time-course and epidemiology of an influenza outbreak, or the potential impact of an intervention measure such as NAI distribution. The mathematical framework introduced here also provides an estimate for the size of the transmitted inoculum. We demonstrate the framework's behaviour using data from ferret transmission studies, and through simulation suggest how to optimise experimental design for assessment of transmissibility. The method introduced here for assessment of mixed transmission events has applicability beyond influenza, to other viral and bacterial pathogens.

## Author Summary

Determining which of two related viruses will spread from human to human more efficiently – e. g. an influenza virus that is treatable with drugs and one that is resistant to them – is important when forecasting the potential impact of an emergent novel virus or developing public health intervention strategies. However, making such measurements of relative transmissibility directly through observation, even using an animal model, is difficult. We have recently developed and published an experimental technique in which an animal is infected with both viruses of interest at once, and then allowed to mix with other animals and so transmit the infection. These experiments provide the necessary data for analysis using the novel mathematical framework that we introduce here. Our mathematical and computational results exploit the power of the experimental system, and allow us to make a quantitative estimate of the relative transmissibility of a drug-resistant influenza virus compared to its drug-sensitive counterpart. Through computer simulation, we demonstrate the wider application of our mathematical technique, and suggest design criteria for future experiments designed to measure the transmissibility of one virus (or other type of pathogen) compared to another.

## Introduction

Under the selective pressure from the host immune system on the influenza haemagglutinin (HA) and the ecological environment, antigenically novel HA ‘drift variants’ of influenza A (IAV) generated by random mutation during replication emerge and circulate in the human population. Intermittent cross-species transmission and/or re-assortment events have the potential to generate antigenically novel (at least for the HA and NA genes) mutant strains of IAV. If transmissible such strains have pandemic potential [1]. As seen in 1918/19, 1957 and 1968, following a brief period of co-circulation with the existing seasonal strain, the pandemic strain typically drives to extinction the previously circulating seasonal variant, to which there is greater prior immunity. However, replacement is not a necessity. In 1977 the existing H3N2 strain continued to circulate after re-emergence of H1N1. In 2010, indications are that the 2009 pandemic H1N1 has replaced the seasonal H1N1 but not the seasonal H3N2 [1], [2].

The use of antiviral drugs has the potential to provide an additional selective pressure on the influenza virus. Between 2007 and 2009 widespread resistance to oseltamivir, the most common neuraminidase inhibitor (NAI), emerged for the H1N1 seasonal IAV [3]–[6], although this replacement event occurred despite the absence of widespread use of oseltamivir in the human population. The mutation in these oseltamivir-resistant H1N1 viruses is a histidine-to-tyrosine mutation at residue 274 (H274Y) in the neuraminidase (NA) gene. The epidemiological observation of replacement indicates that it has a transmission fitness similar to, or greater than, that of the sensitive strain. Other NAI-resistant viruses can have significantly reduced fitness, such that they are unlikely to spread through the population. For example, an arginine-to-lysine mutation at residue 292 (R292K) in the NA gene of seasonal H3N2 viruses confers oseltamivir resistance, but this mutant has not been observed to readily transmit from one host to another [7], [8]. At present, the 2009 pandemic H1N1 strain remains largely sensitive to oseltamivir and has almost entirely replaced the previously circulating NAI-resistant (H274Y) H1N1 strain [2], [3], [9].

Accordingly, during times of co-circulating seasonal strains, emergence of new antigenic seasonal variants, seasonal to pandemic transitions or emergence of drug-resistant strains, assessment of whether or not one strain will out-compete the other and come to dominate the human epidemiology is of relevance to public health planning and response. Key to making predictions on the dynamics of such events is the relative transmission fitness of one strain compared to another. Our group has previously published, in the context of NAI-resistant and NAI-sensitive strains, observations of both growth of mixtures within an animal model and *sustained transmission* of mixtures over multiple host generations of infection [8]. The observation of *transmission of mixtures* allow us to measure the relative transmissibility (“transmission fitness”) of one strain compared to the other. Govorkova et. al. employed a similar experimental design to compare the within-host fitness of an oseltamivir-sensitive and oseltamivir-resistant H5N1 influenza virus pair, but as H5N1 does not transmit readily in the ferret, they were unable to make an assessment of transmission fitness [10]. Duan et. al. have also employed the technique to compare drug-sensitive and -resistant H1N1 pandemic strains [11], probing both contact and respiratory droplet transmission routes.

We introduce a mathematical and statistical framework to capture the key characteristics of *transmission of mixtures*. Our framework allows us to derive a causative-model based estimate of the transmission fitness-cost (or fitness-gain as the case may be) for one strain compared to the other and suggest a method for designing optimised animal experiments. Furthermore, we derive an estimate for the number of successfully infecting units transferred from the donor to recipient, an important measurement for helping parameterize epidemiological models of influenza transmission that consider, for example, the emergence and subsequent spread of NAI-resistant strains [12]–[14].

## Methods

### Motivation

**Figure 1:**
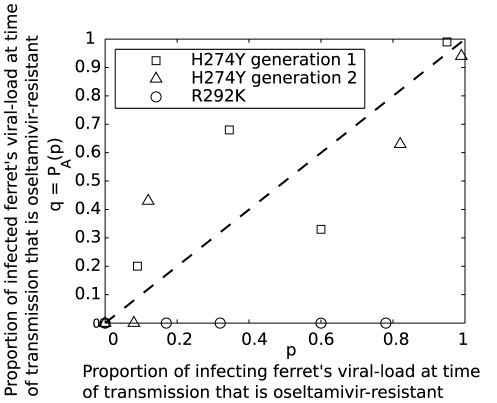
The infectee's mutant (oseltamivir-resistant) proportion, 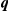, as a function of the infector's mutant proportion, 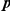. Each point 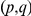 in the figure is a single transmission event between two ferrets. Circles (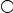) are transmission events for the R292K strain, the mutant known to be severely compromised from previous studies. An experimentally inoculated ‘donor’ infected a first generation ‘recipient’. Squares () and triangles (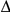) are the first and second generation transmission events for the H274Y strain: donors infecting first generation recipients (squares), and those recipients infecting second generation recipients (triangles). We show the dotted line 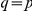 (unit gradient) for reference. Figure (with minor modifications) reproduced from the author's previous work [8] with permission from the American Society for Microbiology.

Figure 1 (adapted from the author's results and reproduced with permission [8]) presents data from a series of contact transmission studies in naïve ferrets. The abscissa shows the proportion of the infecting ferret's viral-load at the time of transmission that is oseltamivir-resistant (the “mutant” strain). The remainder is oseltamivir-sensitive (the “wild-type” strain). The ordinate shows the proportion of the infected ferret's viral-load that is the mutant. Briefly (see [8] for experimental details, and Section *Effects due to data ascertainment limitations* for a detailed discussion of the consequences), the ferrets were swabbed daily, and infection identified by positive real-time RT-PCR. We plot the mutant-proportion in the infected ferret's first positive swab against the mutant-proportion in the infecting ferret's swab from the day prior.

We see that the R292K mutant (circles) does not transmit: no mutant virus is detectable in any of the infected ferrets, the data covering a wide range for the infector's mutant proportion.

For the H274Y mutation we have two generations of transmission. A donor ferret experimentally inoculated with a mixture transmits to a recipient ferret (squares). This ferret is then the infector for the second generation of transmission to another ferret (triangles). The data are consistent with the hypothesis that the infectee's mutant proportion is given by the infector's mutant proportion at the time of transmission.

For convenience, we will always refer to the infector as the donor and the infectee as the recipient. We will use 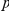 to represent the mutant proportion in the donor and 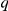 the mutant proportion in the recipient. A data set will be said to have 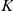 transmission events 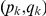, 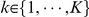.

Three key observations can be made for the H274Y data (squares and triangles in Figure 1):

- The recipient's proportion, 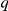, is not constrained to be either zero or one, indicating that there must be more than one virion that successfully enters the recipient and establishes infection. In fact, if we have observed the proportion in the recipient early enough (that is, before significant exponential viral replication has taken place), then the resolution at which the proportions are reported suggests a lower bound on the number of infecting virions. For example, if only three virions were transmitted, then the initial mutant proportion, 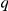, can take only one of four values, seleted from the set 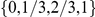. Of course, experimental error will allow for some variation about any one of these four values.
  The fluctuations about the line 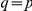 indicate that a stochastic process is at play, either in the transmission event itself or in the early growth phase of the infection within the recipient.
  For one of the second generation transmission events (triangles, Figure 1) the recipient's infection was pure wild-type even though the donor's infection was mixed (data point at 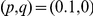, lower left of Figure 1). This indicates that mixed-infections can be lost over time due to the stochastic nature of the transmission and early-replication processes.

We now introduce a model framework to help understand these observations.

### A simple model of mixture transmission

We consider a model with two viral strains, 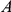 and 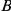. For example, 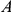 could be the oseltamivir-resistant mutant (either R292K or H274Y) and 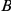 the oseltamivir-sensitive wild-type, as in Figure 1. We introduce the following notation:

- Just prior to transmission, a proportion, 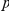, of an infectious *donor* ferret's viral load is of strain 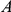 and a proportion 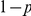 is of strain 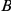.
- Per infectious virion in the donor, a strain 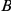 virus is 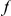 times as likely to be secreted (in a potentially infectious way) than a strain 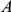 virus. Typically we consider 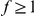.
- Having entered the *recipient* host, the within-host reproductive numbers of strain 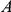 and 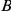 viruses are 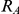 and 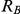 respectively.

#### Stage I: Seeding of virus in the recipient ferret

Suppose that the recipient acquires 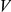 virions from the donor in the transmission event, a mixture of strain 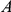 and strain 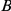. For completeness (and we will return to this in the Discussion), these may be clumped in multiple infectious entities or separate. If clumped, then each infectious entity may itself be a mixture of strain 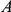 and strain 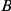 virions. In any case, a straightforward calculation shows that the expected (mean) composition of this load immediately upon infection is as follows:

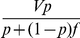
 particles are of strain 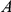.

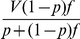
 particles are of strain 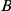.

#### Stage II: Subsequent extinction and growth of surviving virus in the recipient ferret

Lodgement-site specific factors within the host (independent of strain (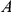 or 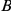)) will prevent all but a proportion 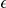 of the 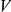 virions from invading a susceptible cell. Of those that do initiate replication, the *expected proportion* of strain 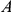 that survive stochastic extinction is 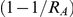, and similarly for strain 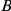 [15].

The surviving progeny are then assumed to undergo exponential growth. We neglect mutation from one strain to another. Some subtleties in the process just described are explored in the Discussion. Thus after 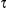 units of generation-time the number of strain 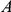 virus particles is:
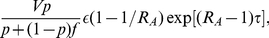
(1)and likewise for the number of strain 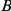 particles. Thus, after some algebra, the *proportion* of particles being strain 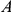 after 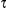 units of generation-time is:
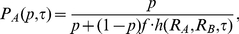
(2)where
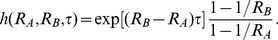
(3)We will usually write 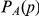 for 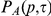 (and similarly 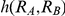) unless we are explicitly considering the generation-time in our analysis. We identify 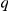 (the ordinate in Figure 1) with 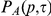.

If 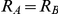 then 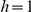. Further, 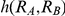 is an increasing function of 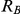 and a decreasing function of 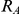, so that if 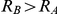 then 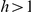, and vice versa. Moreover, if strain 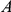 is less secreted than strain 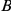 then we have 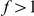. Overall then, having 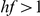 means that strain 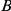 would outgrow strain 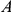 in the recipient, given an even mixture in the donor.

We write 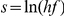 as a *shape parameter* and introduce the functional form:
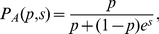
(4)where 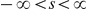.

The shape parameter, 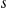, may be interpreted as an overall relative viral fitness of strain 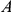 compared to strain 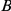, accounting for three factors: (1) secretion from the donor, (2) the initial extinction probability in the recipient and (3) subsequent growth in the recipient.

For example, consider the case in which a drug-resistant mutant (strain 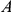) is seeded within a population where the wild-type (strain 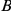) is currently circulating. The strains are antigenically similar (and assumed identical for the sake of this argument) removing any possibility of immunologic selection. In the absense of any drug-selective pressure, if 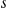 is positive then the wild-type has an advantage over the drug-resistant mutant and so we expect it to continue to dominate the epidemiology. Conversely, if 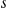 is negative, then the mutant has a fitness advantage and over time would be expected to replace the wild-type strain in the population.

**Figure 2:**
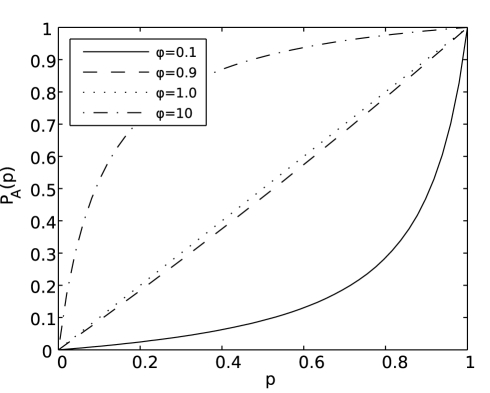
Representative curves for four shape parameters, 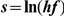. We assume 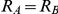 and so 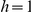. We label the curves by the relative “transmission fitness” of strain 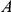 compared to strain 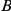, 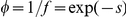. We have 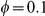 (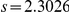) (solid line): strain 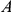
's transmission fitness is 10% that of strain 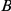, 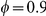 (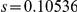) (dashed line): strain 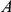
's transmission fitness is 90% that of strain 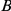, 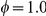 (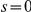) (dotted line): strain 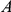 and strain 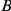 have equal transmission fitness, and 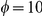 (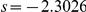) (dash-dotted line): strain 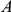
's transmission fitness is 10 times that of strain 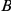.

We have constructed 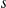 from two components, 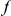 and 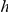, for mathematical convenience and the form has allowed us to interpret the meaning of 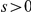 and 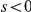. We also find it convenient to decompose the overall measure of relative fitness, 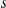, another way. From the three biological factors identified above (labelled (1), (2) and (3)), we consider two components: a within-host “replication fitness” (3) captured by the exponential term in 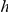 and a relative “transmission fitness” of strain 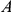 compared to strain 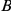, accounting for secretion (1) and extinction (2) given by:
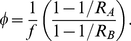
(5)For completeness, although not used herein, we write 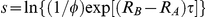. Figure 2 shows four representative curves for the mean behaviour expected from the model, for the simplifying case 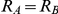, in which 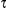 drops out of the model and 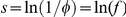.

### Variation in the recipient's strain 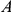 (mutant) proportion

Equation 4 provides the mean estimate for the recipient's strain 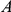 proportion given the donor's strain 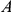 proportion. We wish to derive an estimate for the number of virions transmitted (in a successfully infecting way) and to this end make use of the variation about the mean predicted by equation 4.

We present an approach for the estimation of 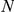, the number of virions transmitted and that initiate infection per exposure event. Note that 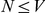 and likely 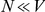, where 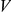 is the number of virions secreted from the donor and that enter into the recipient's airways. The method relies on repeated observations of successful transmission events. Initially we assume that there is no experimental error on the data points and that the number of virions transmitted is fixed across each exposure event.

Before proceeding, we introduce some useful nomenclature. Scripted variables (e.g. 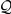) will represent simulated data, non-scripted variables (e.g. 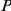), calculations from model equations. Sets will be denoted as 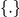, with the 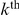 element of a set given by 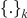.

#### Matching of the residual sum of squares between data and simulation

If we are willing to assume that there is no measurement error for the donor and recipient strain 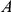 proportions – that is, that the *only* factor leading to the observed deviation of the recipients' proportions from the model predictions is the inherent stochastic nature of the transmission process – then we can make an estimate for 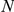.

The usual estimation quantity for a binomial process is the probability of success, given the number of draws. Here, we are interested in the converse: the number of transmitted virions (equivalently, “draws”), given information on the probability of infection per virion (equivalently, “success”).

We consider 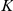 transmission events, each defined by a tuple 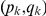, 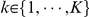, plotted as in Figure 1. First, we determine the best fit model (equation 4), 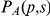 with corresponding estimate, 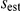, for the shape parameter, and calculate the residual sum of squares, 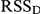, for the data from the model:
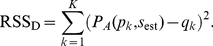
(6)


Having determined the RSS of the data given the model, 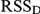, we now ask: What inoculum size, 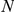, when used to generate synthetic data from the best-fit model with shape parameter 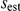, gives an RSS closest to 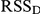
?

As a function of 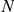 (with 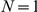 up to 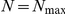, with 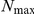 some sufficiently large integer), we simulate the random draw of 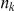 particles of strain 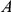 from 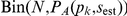, where 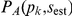 is identified as the probability of selecting a strain 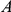 virion from the 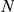 transmitted virions. We obtain a simulated data set 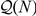, with 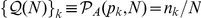.

We calculate the residual sum of squares, 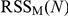, for the simulated data (compared to the *model*) as a function of 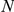:
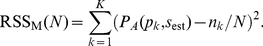
(7)We then choose the minimum difference in RSS as our estimate for 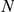:
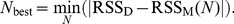
(8)


Finally, we repeat, scanning over 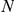 for many generated synthetic data sets 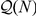, on each occasion selecting that 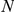 with minimum difference in RSS as our estimate 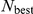. We obtain an empirical distribution of best fit 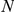
_s_, from which we can calculate an average, 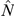, and variance.

### Effects due to data ascertainment limitations

Until now, we have implicitly assumed that an observation 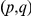 is without error. However, experimental limitations, including animal ethics principles and practicalities within the laboratory environment, introduce uncertainty into any measurement of a transmission event. Three sources of experimental error are of potential significance:

1. The mutant and wild-type viral loads are measured using a real-time RT-PCR (rRT-PCR) assay.
2. The animals are swabbed at periodic intervals, introducing a sampling window for the time of infection. In the H274Y experiments used here, and published in full by Hurt et. al. [14], swabs are taken daily.
3. In the experimental design used by Hurt et. al. [14] there are two donor ferrets housed with a single recipient ferret in the first generation of transmission (squares in Figure 1).

Each needs to be assessed and if necessary accounted for when performing the proposed analysis on a new data set. We address each issue in turn, in a semi-quantitative way, using the H274Y data to illustrate the issues.

#### The rRT-PCR assay

The strain 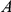 (mutant) *proportion* as measured using the rRT-PCR assay is accurate to within 1–3 percentage points across experimental repeats [8]. Furthermore, per assay, the result is extremely accurate as the following analysis reveals. Per cycle in the rRT-PCR assay, the amount of product doubles (prior to saturation). The cycle-threshold (Ct) is defined as the number of cycles required for the product (marked by a fluorescent signal) to exceed a specified threshold. A lower Ct value indicates a larger quantity of product in the sample as fewer doubling cycles are required to reach the threshold. The strain 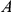 proportion is determined from the differences in Ct values for the strain 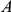 and strain 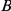 strains as follows:
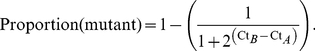
(9)The Ct values are measured to two decimal places in the experiments reported by Hurt et. al. [8]. A simple numerical calculation probing the worst-case scenario (attempting to maximise (or minimise) the difference 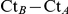) shows that with this accuracy on the recording of the Ct values, the mutant proportion as calculated using this formula and reported by Hurt et. al. [8] would be unchanged (at two significant figures for the proportion) in almost all cases. A few of Hurt et. al. 's reported values could, in the worse case, fluctuate by just 1 percentage point.

In summary, based on repeatability of rRT-PCR assay, we may expect a small (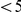) percentage point error to be introduced.

#### Periodic sampling for infection

**Figure 3:**
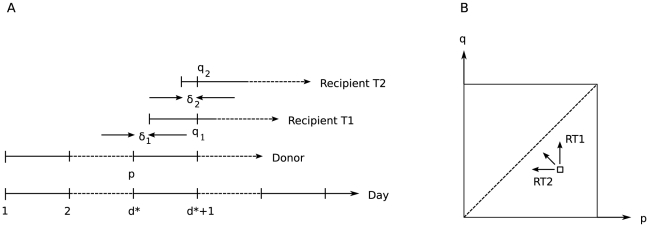
Bias due to periodic sampling for infection. **A**: A sketch of two hypothetical transmission events occurring at some time between days 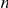 and 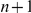. Measurements of viral load are taken from the donor and recipients on days 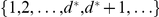. The transmission from Donor to Recipient T1 occurs at time 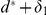. The transmission from Donor to Recipient T2 occurs at time 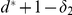. For both events we record the Donor's strain 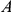 proportion, 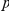, at time 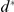 and the recipient's strain 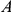 proportion, 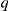, at time 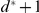. **B**: A sketch of the recorded data point (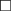) assuming that systematic processes dominate and that the strain 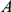 proportion drops over time due to a reduced within host reproduction fitness of the strain 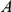 strain. The arrows 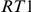 and 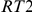 for recipients 1 and 2 respectively in **A** indicate the direction in which the true infection event lies in the 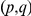-plane for small 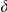. The diagonal arrow shows the “correction-direction” for an infection event occurring at an arbitrary time point between times 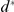 and 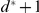. Note that because the process of viral growth within the host is non-linear, the relative shift horizontally and vertically cannot be related directly to the time of infection between 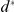 and 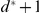. If the strain 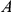 virus had a higher within-host replication fitness than the strain 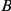, then the arrows would be reversed. If stochastic processes dominate, the systematic effect may be overwhelmed, leading to reduced bias due to the sampling window.

The second issue proves more interesting. With a daily sampling window for swabbing the animals, we potentially introduce a systematic bias into the reported paired 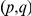 transmission results. Figure 3**A** represents a donor (“Donor”) infected at day 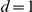 and infecting a recipient ferret during the time window 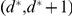. We show two *different* – that is non-concurrent and non-interacting – hypothetical transmission events to a recipient, termed either “Recipient T1” or “Recipient T2” as appropriate. All swabs for all animals are taken at days 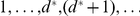. A transmission event occurring in the time window 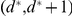 will be recorded as 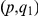 or 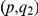 for Recipient T1 or T2 respectively, and by construction, both of these pair-values will be the same. However, as shown in Figure 3, Recipient T1 was infected just after time point 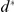 while Recipient T2 was infected just prior to time point 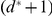. Figure 3**B** plots a square at location 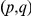 as recorded in the experiment. Assuming that the within-host transmission fitness of the mutant is less than that of the wild-type (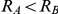), and that stochastic processes *do not* overwhelm this systematic effect of a reduced replication rate for the mutant, the arrows (RT1 for Recipient T1 and RT2 for Recipient T2) show the direction in which the true transmission pair 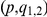 would lie. We argue as follows:

- For “Recipient T1”, as 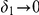, the measurement for 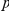 is exact, while the measurement for 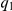 (recorded at time 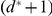) is a full time-unit too late. The correct value for 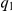 is that which would have been recorded just after time point 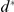. Assuming 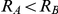, this will be a larger value. We can only infer this value by fitting a model to the mutant proportion over time and back projecting as the swab for Recipient T1 at time 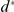 was by definition negative.
- For “Recipient T2”, as 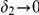, the measurement for 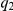 is exact, while the measurement for 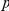 (recorded at time 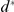) is a full time-unit too early. The correct value for 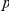 is that recorded at time point 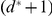. Assuming 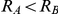 this will be a smaller value.

The diagonal arrow shows the “correction-direction” for an infection occurring at an arbitrary infection time during the time window 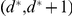 for the case when 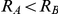. Note that if the mutant were more fit than the wild-type (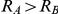), the bias (arrows) would be in the opposite direction. For equally fit viruses, there is no bias introduced due to the measurement period.

The preceding argument assumed that the within-host dynamics proceeded in a systematic noise-free way, with the measurement for 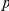 or 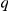 *over time* being well behaved and well predicted by the difference between 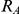 and 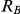. If, however, stochastic processes overwhelm the systematic within-host differences between strains, then the potential bias introduced due to the sampling may not be evident in the data. The revised mutant proportion (in either the donor or recipient) may be either larger or smaller than the reported value. In this case examination of the experimental data on a case-by-case data is necessary and then only “Recipient T2” type events may be reliably assessed as without data at time point 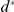 for a “Recipient T1” event no revised estimate for 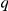 is available.

#### Multiple donor ferrets

The third issue arising from the experimental design – and one which is easily avoidable in future experiments – is that we do not know which donor ferret was responsible for the transmission event to the first recipient. The mutant proportion for the donor shown in Figure 1 is the average of the mutant proportions in the two donor ferrets at the sample time directly proceeding confirmation of infection in the recipient. As it may be that the mutant proportion as measured each day in any given ferret is subject to substantial fluctuations, and that each donor ferret has a unique experience, there is significant potential for this source of error to be large.

For the five relevant transmission events in the H274Y experiment we have the following donor 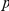 pairs (not 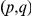 pairs) at the time of transmission: (0.00,0.00), (0.09,0.09), (0.38,0.31), (0.56,0.64) and (0.96,0.94). In this small sample, we have up to a 4 percentage point difference between the average across donors and individual recorded values.

In general, there is scope for significant uncertainty due to multiple potential sources of infection and the statistical inference should take this into account.

### Synthetic data for simulation-estimation studies for algorithm testing and experiment design

With only a limited amount of data available from ferret experiments, we use results from simulation-estimation studies to optimize future experimental design and make predictions on the likely precision of estimates for shape parameters from future experimental observations.

Our simulation model has four free parameters:

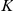
, the observed number of transmission events. We consider 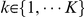;

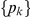
, the set of assumed values for the donors' strain 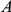 proportion at the times of transmission;

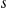
, the assumed shape parameter, which if we assume 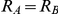 and so 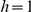, is determined by the relative transmission fitness, 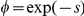, of strain 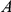 compared to strain 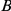; and

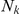
, the assumed number of virions transferred from the donor to recipient in the 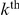 transmission event. Here we will assume 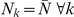 but in general may consider 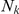 to be sampled from 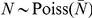, where 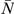 is the expected number of virions transferred from the donor to recipient in a transmission event.

Synthetic data, 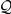 with 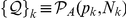, for 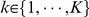 transmission events, each from a donor transmitting 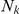 virions with strain 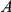 proportion 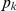 at the time of transmission, is generated as follows.

First, we specify the shape parameter 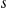 for equation 4, defining 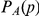. If 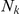 is a random integer drawn from 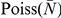 and 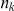 is a random integer drawn from 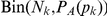 then our synthetic data, 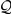, are given by (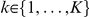):
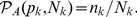
(10)If 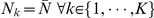 then 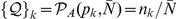.

### Estimation of 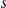 and 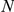


We estimate the shape parameter, 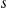, by fitting the model (equation 4) to either real or synthetic data, by application of non-linear least squares regression (routine nlinfit in MATLAB R2010a). We estimate the number of virions, 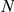, using equations 6 through 8.

## Results

We now present results of applying our model to both data from ferret experiments and simulation studies. We re-iterate that our use of data from the contact transmission study [8] is to demonstrate proof-of-principle, and that our results cannot be seen as definitive for the particular influenza virus pair used in that experiment, due to the small number of observed transmission events and some of the limitations as discussed earlier in Section *Effects due to data ascertainment limitations*.

### Estimates for 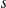 (and so 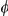) and 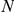 from the H274Y ferret experiments

**Figure 4:**
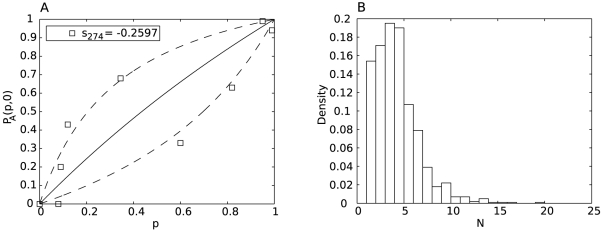
Model fit (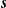 and 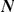) for the H274Y data. **A**: Best fit (sold line) and 95% confidence interval (dashed lines) model curves for the H274Y data (squares) shown in Figure 1. **B**: Histogram of the best fit number of virions, based on 1,000 sets of simulation scans over 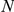. The mean is 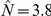 and variance is 5.9.

Applying our model equation 4 to the data for the H274Y mutation shown in Figure 1 (and assuming the data are recorded without error – see below for a generalised analysis) provides an estimate for the shape parameter of 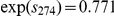 with a 95% confidence interval of (0.259, 2.30), calculated under the assumption of i.i.d Gaussian residuals, which is not strictly true. Assuming 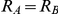 – which is a valid approximation given the within-host analysis of this data set [8] – the corresponding estimate for the relative transmission fitness for the H274Y mutant, in a direct-contact scenario, is 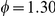 (0.44,3.9). We predict that an average 3.8 virions were responsible for each transmission event, with a variance for our estimate of 5.9, but we caution that this estimate is based on an assumption of data recorded without error. Figure 4**A** shows the model fit to the H274Y data and Figure 4**B** shows a histogram of the best 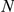
's.

#### Assessment of effects due to bias in the sampling window

We now account for the three sources of error discussed in Section *Effects due to data ascertainment limitations*. Firstly, the rRT-PCR assay introduces a small (1–3 percentage point) uncertainty into our data (both 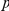 and 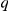). Secondly, as reported on in detail by Hurt et. al., data were collected at daily intervals from the ferrets [8]. An examination of that data indicates that stochastic, rather than systematic, effects dominate. The mutant (strain 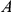) proportion measured at adjoining time points shows significant variation over and above the systematic reduction expected from the marginally reduced within-host fitness of the mutant. In consequence, while we cannot make an assessment of the “Recipient T1” error (see below), we can still make a direct estimation of the “Recipient T2” error from the daily mutant proportion data. We estimate (in a semi-quantitative way) that we may introduce up to a 10–15 percentage point error in 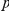 due to this stochastic variation, but it may be far less for any individual data point. Conservatively, in the absence of information to inform the “Recipient T1” error we allow for an additional 10 percentage point error on 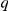. Finally, we can also assess the possible modifications to model based inferences due to averaging across the two donor ferrets for the first transmission event in each experiment. We estimate that the variation due to this is of the order of 5–10 percentage points, again an influence on 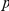 and variable from data point to data point. The data in [8] allows us to make a direct estimate of this on a transmission-event by transmission-event basis.

Listing the transmission events shown in Figure 4**A** from left to right on the 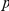-axis, Table 1 shows our estimate for the plausible range for that tuple's 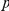 and 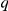 values. For clarity, we list all entries as percentage points, rather than proportions.

**Table 1:** Assessed credible range for the H274Y transmission data tuples 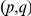.

Transmission event 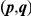	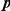 , assessed interval for 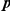	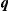 , assessed interval for 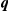
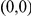 *	0, [0,0]	0, [0,0]
(8,0)	8, [6,19]	0, [0,0]
(9,20)	9, [7,32]	20, [12,28]
(12,43)	12, [10,36]	43, [35,51]
(34.5,68)	34.5, [3,40]	68, [60,76]
(60,33)	60, [44,73]	33, [25,41]
(82,63)	82, [64,84]	63, [55,71]
(95,99)	95, [92,100]	99, [91,100]
(99,94)	99, [97,100]	94, [86,100]

The experimentally reported data tuples 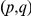 (shown as a percentage for clarity) for each of the 10 transmission events shown in Figure 4**A** (column 1) and an estimate for the uncertainty in 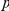 (column 2) and 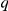 (column 3) based on the three sources of error as discussed above and in the Methods.

*Note that there are two transmission events at (0,0).

**Figure 5:**
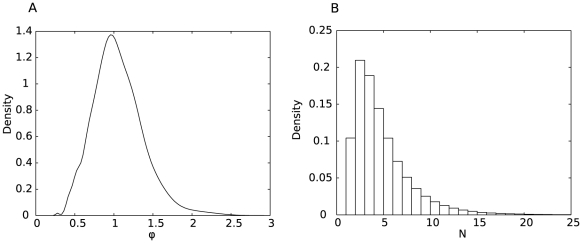
Parameter estimation for the H274Y data when taking experimental data uncertainty into account. **A**: The transmission fitness, 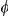. **B**: The transmitted inoculum size, 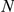.

To make an assessment of the influence that these uncertainties may have in estimating the relative transmission fitness of strain 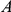, we take a simulation approach. We generate 1,000 equivalent synthetic data sets in which, for each of the 10 observed transmission events, 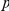 and 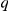 for the tuple are drawn independently and randomly from the uniform distribution over the range identified in Table 1. For each of the 1,000 synthetic data sets we fit the model, recording the best fit shape parameter 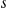. Reporting the relative transmission fitness for convenience, we recover 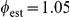 (0.51, 1.73), where the 95% confidence interval is empirically determined from the distribution of recovered best fit values for 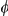 over the 1,000 simulations. Similarly, to determine the number of transmitted virions, 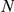, we apply equations 6–8 to each synthetic realisation, and combine the empirical distributions, obtaining a mean of 4.3 and variance of 9.8. Figure 5 shows the results.

Comparing these results to those in Figure 4, the key difference is an extension in the tail of the distribution for the number of virions transmitted. Over the 1,000 samples of the synthetic data, some will fall closer to the model curve, and so the residual variation, attributed to the stochastic nature of the transmission process, will be smaller. The consequence is a larger estimate for 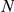. A minority of synthetic data sets based on the distributions for 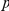 and 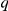 as shown in Table 1 have this reduced residual variance, while the mean is almost unchanged, suggesting that the method first presented in which the three sources of experimental error are ignored is valid, at least for this data set.

### Estimation of 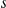 and 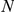 from simulations

#### Simulated scenarios

For reporting convenience we here assume that 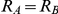 and present the relative transmission fitness, 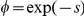 rather than the shape parameter, 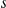. If 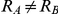 one needs to make an estimate of the reproduction numbers by other means to separate out the within-host and transmission comfponents of the overall relative fitness of strain 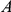 compared to strain 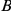.

To assess the possible variation in observed data and implications for inference, we generate 1,000 simulated data sets for each specified combination of parameters shown in Table 2. We then extract the best fit shape parameter, 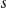 and number of transmitted virions, 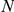, for each simulated data set. Determination of the average best fit shape parameter and average best fit number of virions is done numerically in order to avoid unnecessary assumptions of normality when estimating confidence intervals.

**Table 2:** Simulation scenarios for model validation.

Simulation set	( 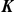 , 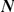 , 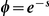 )	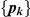	Comments
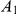	(10, 4, 1.05)	Evenly spaced from 0.05 to 0.95.	Estimates from the H274Y experiment data.
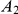	(10, 4, 0.9)	Evenly spaced from 0.05 to 0.95.	As per 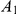 , 10% compromised mutant.
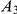	(10, 4, 0.8)	Evenly spaced from 0.05 to 0.95.	As per 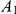 , 20% compromised mutant.
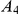	(10, 4, 0.5)	Evenly spaced from 0.05 to 0.95.	As per 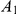 , 50% compromised mutant.
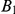	(30, 4, 1.05)	Evenly spaced from 0.05 to 0.95.	As per 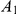 , three times as many transmission events.
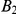	(30, 4, 0.9)	Evenly spaced from 0.05 to 0.95.	As per 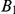 , 10% compromised mutant.
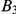	(30, 4, 0.8)	Evenly spaced from 0.05 to 0.95.	As per 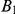 , 20% compromised mutant.
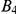	(30, 4, 0.5)	Evenly spaced from 0.05 to 0.95.	As per 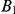 , 50% compromised mutant.
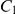	(90, 4, 1.05)	Evenly spaced from 0.05 to 0.95.	As per 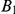 , three times as many transmission events.
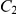	(90, 4, 0.9)	Evenly spaced from 0.05 to 0.95.	As per 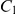 , 10% compromised mutant.
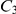	(90, 4, 0.8)	Evenly spaced from 0.05 to 0.95.	As per 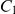 , 20% compromised mutant.
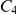	(90, 4, 0.5)	Evenly spaced from 0.05 to 0.95.	As per 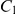 , 50% compromised mutant.
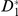	(30, 4, 1.05)	Evenly spaced from 0.4 to 0.6.	As per 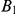 , observations clustered around central values for 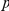 .
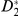	(30, 4, 1.05)	Evenly spaced from 0.05 to 0.25 and 0.75 to 0.95.	As per 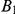 , observation clustered near the boundary values for 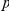 .
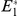	(10, 50, 1.05)	Evenly spaced from 0.05 to 0.95.	As per 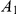 , lower variability (increased 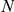 ).
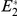	(30, 50, 0.5)	Evenly spaced from 0.05 to 0.95.	As per 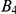 , lower variability (increased 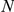 ).

For each simulation 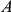 through 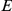, we specify four parameters: 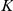, the number of transmission events observed, 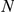, the number of virions transmitted in each transmission event, 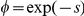 (assuming 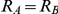) the relative transmission fitness of strain 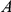 compared to strain 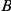, and the set 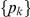, the mutant proportion in the donors for each transmission event 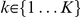. *Results for simulations 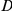 and 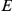 are presented in Text S1.

Scenario 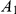, based on the H274Y data, provides the reference point for all other scenarios. Scenarios 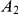, 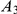 and 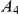 explore a reduction in the relative transmission fitness to 0.9, 0.8 and 0.5 respectively, while keeping the number of transmission events, 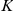, fixed at 10. In scenarios 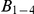 the number of observed transmission events is increased by a factor of three to 30, while all other biological and experimental parameters are kept unchanged relative to the 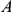-scenarios. Scenarios 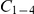 examine a further increase in the number of observed transmission events to 90. Scenarios 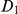 and 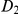 explore if more limited strain 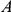 proportion ranges in the donors (the set 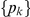) may change the precision of the estimates for 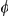 and/or 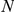. Scenarios 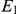 and 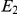 examine hypothetical mutants which transmit a larger number of virions (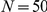) in a typical transmission event.

#### Parameter estimates for scenarios

**Figure 6:**
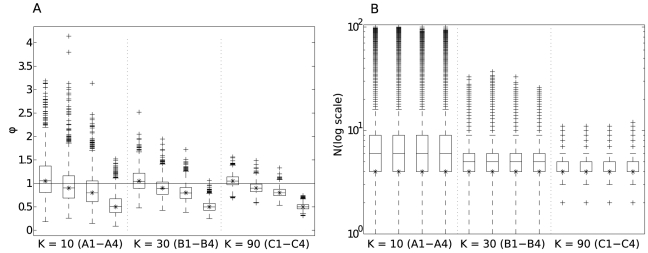
Recovered estimates for 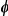 and 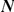 for simulations 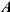, 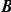 and 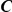. The simulations are described in Table 1. **A**: The transmission fitness, 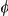. **B**: The transmitted inoculum size, 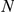. Note the log scale for the estimate of 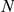. Each boxplot shows the median, 25th and 75th centiles with tails extending to the upper and lower adjacent values and outliers shown as crosses. The asterisk marks the true value used in the simulation. The dashed vertical lines are a visual aid to separate simulations 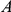, 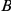 and 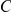. The horizontal line in **A** shows a relative transmission fitness of 1. Note that the median and 25th centiles for the estimated 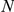 are commensurate for simulation 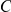. The uncertainty in estimates for both 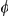 and 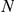 reduces with increasing number of transmission events (e.g. compare scenarios 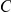 to 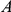 in both panels). For a given number of transmission observations (fixed 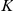), the estimate for 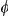 is more constrained for lower true values of the transmission fitness (e.g. compare 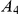 (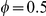) to 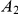 (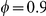) in **A**). The estimate for 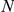 does not improve for lower true (and so predicted) values of the transmission fitness (e.g. compare 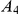 and 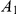 in **B**).

Figure 6 shows a summary of the recovered estimates for the relative transmission fitness, 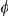 and the transmitted inoculum size, 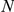, for the 12 simulations 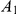 through 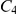 listed in Table 2. Results for scenarios 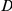 and 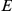 are presented in Text S1. Each boxplot shows the median, 25th and 75th centiles with tails extending to the upper and lower adjacent values and outliers shown as crosses. The asterisk marks the true value used in the simulation. Note that a log scale has been used for 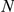 due to the extended right tail. Text S1 contains detailed results for all simulation runs.

Figure 6**A** shows that we have an unbiased estimator for the relative transmission fitness, 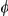. As expected, the uncertainty in the estimate for 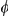 reduces with increasing number of observations (e.g. compare simulations 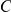 to 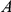). To exclude 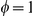 from the estimate (of key relevance to public health) a larger number of transmission observations need to made when the true value of 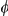 is closer to 1.

We have an accurate, although slightly biased estimator for the transmitted inoculum size, 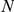. The mean and median estimates for 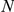 are always larger than the true value, while the mode is unbiased (see Text S1 for more details). Figure 6**B** indicates that the bias reduces as the number of observed transmission events, 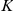, increases. If the true value for the transmitted inoculum size is greater, the estimator is far less able to exclude very large values for 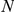 (see Text S1, simulations 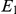 and 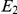)

## Discussion

The relative transmissibility of one strain of influenza compared to another is important in a number of different contexts: seasonal/seasonal interactions, seasonal/pandemic interactions and NAI-resistant/NAI-sensitive interactions. Given the competitive mixtures animal model paradigm is applicable in all three contexts, the mathematical framework presented here has wide applicability in influenza research.

Our results indicate that application of equation 4 and equations 6–8 to transmission data from mixed infection studies allows estimation of both the transmission fitness of a new strain of interest (strain 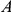) relative to that of the existing wild-type (strain 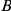) and the transmitted inoculum size. Our consideration of the uncertainties inherent in the data source due to experimental procedures indicate that the method is robust to these issues, although we caution that any application of the method should consider each source of uncertainty in the data on its merits.

The simulation studies show that the method accurately recovers the relative transmission fitness, 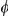 of strain 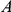 compared to strain 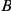. As expected, the closer the true value of 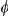 is to unity, the greater the number of transmission events that need to be observed to exclude 1 from the 95% confidence interval for 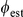. For recovering the number of virions transmitted and successfully initiating infection, the mode of the distribution is unbiased, while the mean and median are consistently slightly larger, due to the extended right-tail of the distribution. It follows that this method can be used in experiment design to ensure that studies are sufficiently powered to provide estimates of a specified precision for the transmission fitness.

In our proof-of-principle analysis of the H274Y data set [8], for both simplicity and because the data indicate that it is a good approximation, we have assumed that 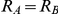 and so 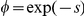 in equation 4. If the within-host replication fitnesses of the two strains were not equal, then an estimate of the effective reproduction numbers (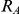 and 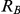) and the generation time, 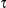, for the within-host dynamics would be necessary to correctly infer the relative transmission fitness, 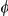, from the estimate of the shape parameters, 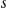. Furthermore, a simulation analysis allowing for uncertainty in infection time (along the lines of the argument laid out in Section *Effects due to data ascertainment limitations*) would also be necessary.

Our method does not explicitly require knowledge of the immune status of the ferrets, the shape parameter providing information on the effective fitness of strain 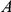 compared to strain 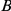 given the natural history of infection and/or vaccination of the animals. Differences in immunity to strains 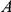 and 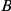 manifest as changes in the effective reproduction numbers (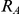 and 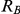). Consequently (as just discussed above), to determine the relative transmission fitness, 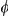, one must obtain independent knowledge of 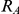, 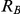 and 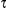. This may be achieved by conducting an analysis of the within-host dynamics of competitive-mixtures infection in naïve and non-naïve animals. Estimation of the within-host reproduction number is a key focus of the rapidly developing within-host influenza dynamics literature [16]–[20].

Our method indicates a number of potential improvements in competitive-mixtures experimental design. Firstly, the use of multiple donor ferrets – introduced by Hurt et. al. as a “reliability” factor in the face of uncertainty of infection following inoculation – introduces unnecessary complications and should be avoided given that experimental inoculation has been shown to reliably result in infection. Secondly, regular sampling is essential so as to tie down as closely as possible the strain 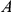 proportion in both donor and recipient at the time of transmission. This is of particular importance if 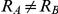 as just discussed. An alternative way to more accurately ascertain 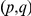 may be to introduce recipient ferrets to the infectious donor for controlled and limited times. Such a design has the additional appeal of allowing determination of the donor's time-dependent infectivity, of particular relevance to public health planning in influenza (see, e. g. [21]).

We see scope for improvement to the mathematical framework in a number of ways. Firstly, the number of virions transmitted and successful at initialising infection in the recipient was assumed constant (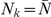 in equation 10). Clearly this is not strictly true and it would be possible to probe the effects of the Poisson nature of the process, from transmission event to transmission event, in more detailed simulation studies.

We have also assumed that virions are deposited and die or replicate independently of one another. However, it is plausible that virions and indeed mixtures of strain 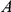 and strain 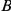 virions, clump together in the process leading to their secretion from the donor and transmission to the recipient, for example, in droplet or aerosol particles. Our analysis of the H274Y contact transmission data indicates that 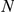, the number of virions that successfully establish infection is small, although the result has not been established for aerosol or droplet transmission. While we are not aware of any direct experimental evidence from influenza studies allowing comparison, Abrahams et. al. report that for HIV infection, 78% of infections involved a single variant (using their terminiolgy, a single “infectious unit” and so we tentatively suggest a single virion), while the remaining infections were established by between 2 and 5 (median 3) “infectious units” [22]. While we estimate that 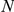 is small, it is entirely plausible that the total number of potentially infectious virions transmitted, 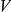, may be somewhat or perhaps even much larger (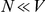). Each transmitted entity (or “unit”), itself containing perhaps multiple virions, may either lodge at a site compatible with replication or not. Of those that do (essentially the proportion 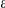 in equation 1), local within-host competitive processes between strain 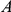 and 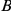 virions will then come into play. If multiple entities are successful in establishing local sustained growth within the host, the eventual establishment of a mixed-infection may be due to a hybrid of the heterogeneity in mixtures across entities, and the local within-host processes at play as each entity establishes infection. How to probe such possibilities, both experimentally and theoretically, presents as an interesting future research opportunity.

The third avenue for improvement concerns accounting for re-assortment of strains 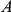 and 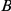 during the within-host dynamics, in both the donor(s) and recipient(s). For the H274Y data, this is not of concern, due to the similarities between the two strains [8], but more general experiments, say comparing a new pandemic strain to an existing seasonal strain of a different sub-type, would need to be analysed accounting for the additional complexity.

With the limited data available from the H274Y experiment, we were unable to meaningfully constrain the relative transmission fitness of the NAI-resistant strain (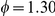 (0.44, 3.9)). We also caution that the result is for contact transmission, and there are indications that respiratory droplet and/or aerosol transmission experiments may yield different results, as demonstrated recently by Duan et. al. [11]. However, our simulation analysis, which can be considered as a computational power calculation, indicates that we should be able to identify a relative transmission fitness – in either contact or droplet transmission studies – of 0.9 or less with a large but not prohibitive number of ferrets (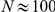). It should be kept in mind that our analysis can be performed on pooled data from multiple experiments, each with other primary end-points of interest, as long as those experiments do not probe interventions that may be expected to modify the *relative* transmissibility of one strain compared to the other (such as immune changes, vaccination etc.).

Finally, the method presented here is applicable to pathogens other than influenza as any series of observations of transmission of mixtures may be represented as in Figure 1. The most critical factor requiring thought will be the within-host dynamics of the two strains, and in particular, appropriate consideration of any competitive or synergistic effects due to co-infection.

## Supporting Information

Text S1 Results of simulation studies. We present detailed results from the simulation studies outlined in Table 2.
(PDF)
