# Supplementary material for: A Mathematical Framework for Estimating Pathogen Transmission Fitness and Inoculum Size Using Data from a Competitive Mixtures Animal Model
Source: PLoS Comput Biol. 2011 Apr 28;7(4):e1002026. doi: 10.1371/journal.pcbi.1002026 (PMC3084214; doi:10.1371/journal.pcbi.1002026)
Supplement: Text S1 — Results of simulation studies. We present detailed results from the simulation studies outlined in Table 2. (PDF) [file pcbi.1002026.s001.pdf]

A mathematical framework for estimating viral transmission fitness and inoculum size using data from a competitive mixtures animal model. Text S1: Results of the simulation studies

Figure 7 shows the recovered estimates for ϕ and N for simulation scenarios D and E described in Table 1 in the main text. Simulations D_1 and D_2 (when compared to both each other and simulation A_1) demonstrate that we obtain marginally tighter estimates for ϕ and N by clustering the samples for the donor strain A proportion, p, around either the centre or extreme values. Simulations E_1 and E_2, where the transmitted inoculum is increased (to 50, up from 4), confirm the expected increase in precision for ϕ due to the lower variability in outcome from the binomial selection process. There is a substantial decrease in precision for the estimate of N.

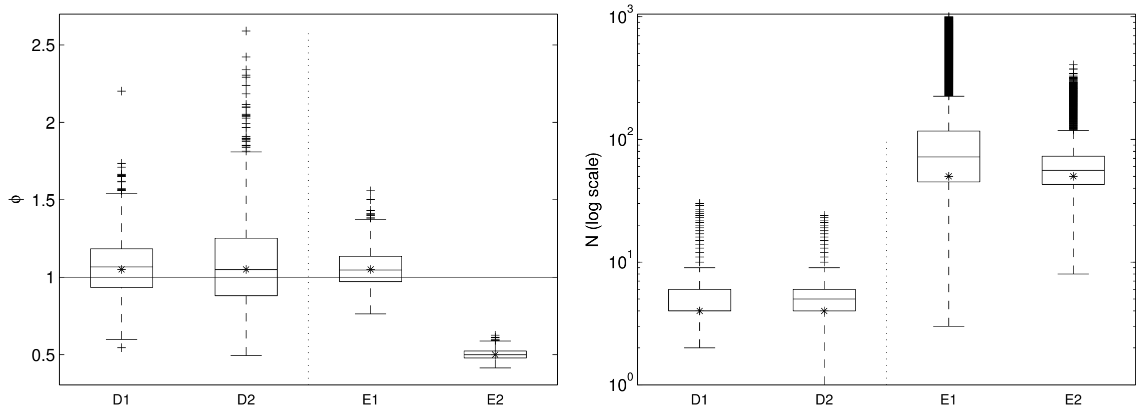

(a) The relative transmission fitness, ϕ

(b) The transmitted inoculum size, N

Figure 7: Recovered estimates for ϕ and N for simulations D and E described in Table 2 in the main text. Note the log scale for the estimate of N. Each boxplot shows the median, 25th and 75th centiles with tails extending to the upper and lower adjacent values and outliers shown as crosses. The asterisk marks the true value used in the simulation. The dashed vertical line is a visual aid to separate simulations D and E. The horizontal line in Figure 7(a) shows a relative transmission fitness of 1. Simulations D_1, D_2 and E_1 should be compared to A_1 and E_2 to B_4.

Figures 8 through 12 present the detailed results for each simulation scenario described in Table 2 in the main text. Each figure shows the numerically determined probability density for the recovered relative transmission fitness ϕ (left) and a histogram for the recovered transmitted inoculum size, N (right). We report the mean and 95% confidence interval for ϕ, and the mode, mean and standard deviation for N.

Evident in Figure 6 in the main text and Figure 7 here, there is a minor bias in the estimate for N. This can be understood by considering $\mathrm{RSS}_\mathrm{M}(N)$ in Equation 7 in the main text. $N = N_\mathrm{true}$, where N_true is the true value of the transmitted inoculum size, is the N *most likely* to generate the minimum difference between RSS_D and $\mathrm{RSS}_\mathrm{M}(N)$ and so the mode of our distribution is accurate. For values of $N < N_\mathrm{true}$ the residual must be too large, while for $N > N_\mathrm{true}$ the residual, while expected to be too small, may be too large. It follows that a slight right bias will be found in the histogram for N.

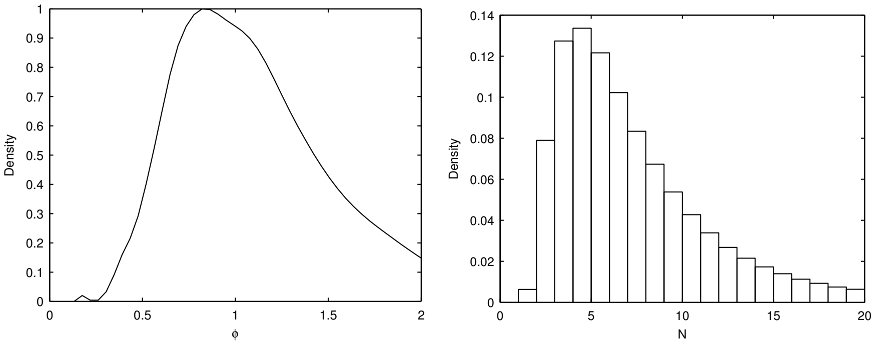

(a) Simulation A_1: $\phi_{\text{est}} = 1.14$, (0.47, 2.40), mode$(N_{\text{est}}) = 4$, mean$(N_{\text{est}}) = 7.3$, $\sigma(N_{\text{est}}) = 5.5$

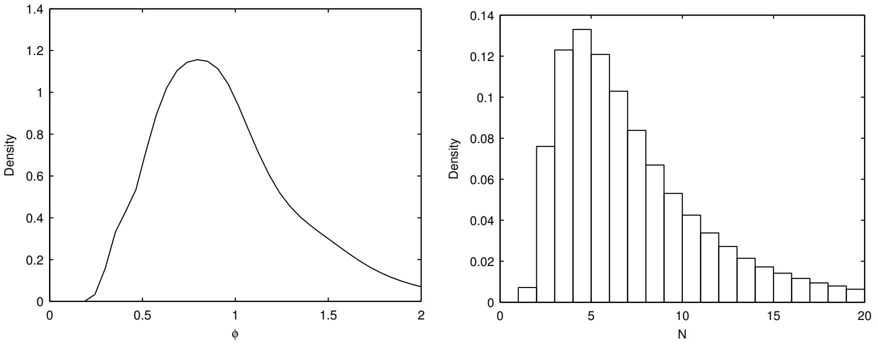

(b) Simulation A_2: $\phi_{\text{est}} = 0.98$, (0.38, 2.02), mode$(N_{\text{est}}) = 4$, mean$(N_{\text{est}}) = 7.5$, $\sigma(N_{\text{est}}) = 6.2$

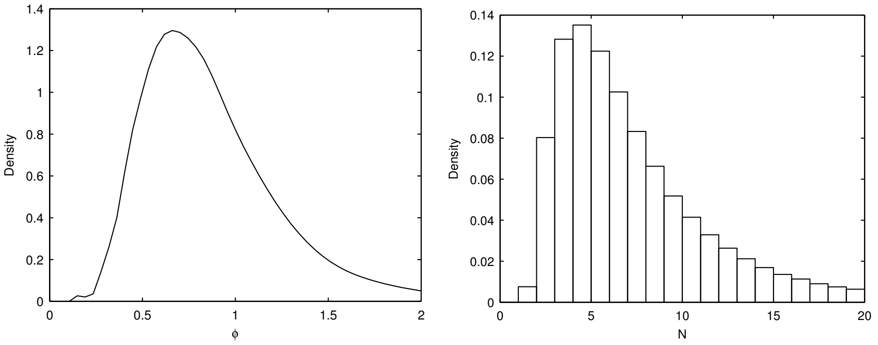

(c) Simulation A_3: $\phi_{\text{est}} = 0.87$, (0.36, 1.83), mode$(N_{\text{est}}) = 4$, mean$(N_{\text{est}}) = 7.3$, $\sigma(N_{\text{est}}) = 5.5$

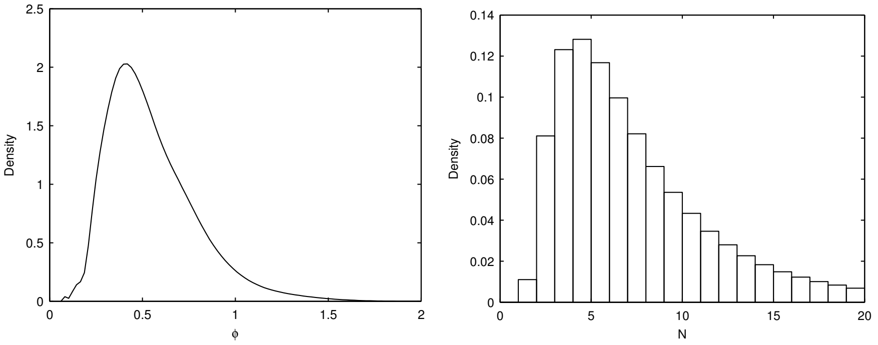

(d) Simulation A_4: $\phi_{\text{est}} = 0.55$, (0.22, 1.09), mode$(N_{\text{est}}) = 4$, mean$(N_{\text{est}}) = 7.4$, $\sigma(N_{\text{est}}) = 5.7$

Figure 8: Simulation A: Fits to ϕ (left) and N (right)

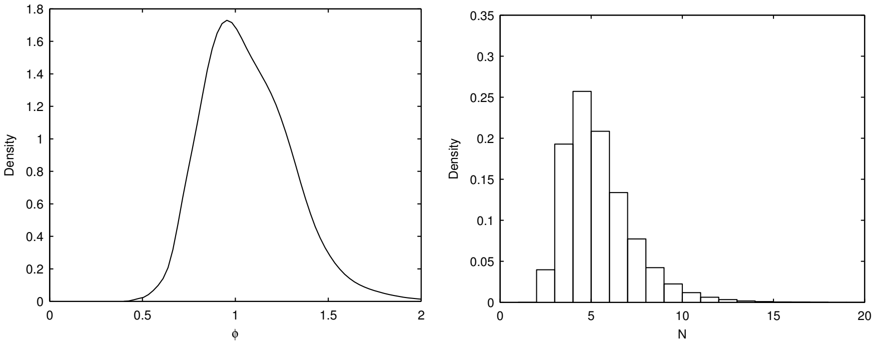

(a) Simulation B_1: $\phi_{\text{est}} = 1.07$, (0.70, 1.57), mode(N_{est}) = 4, mean(N_{est}) = 4.9, $\sigma(N_{\text{est}}) = 1.9$

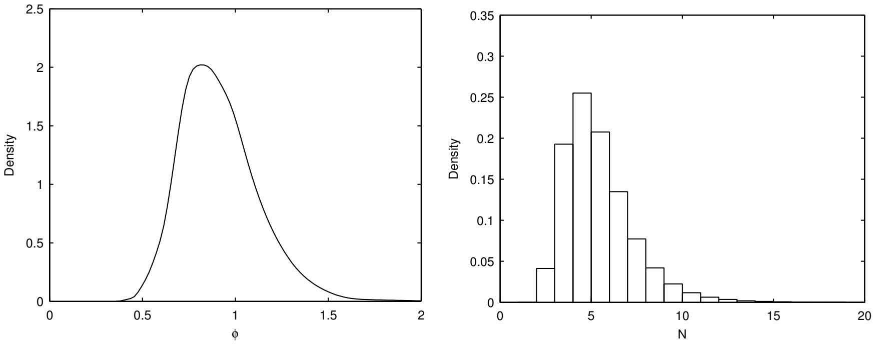

(b) Simulation B_2: $\phi_{\text{est}} = 0.91$, (0.58, 1.37), mode(N_{est}) = 4, mean(N_{est}) = 4.9, $\sigma(N_{\text{est}}) = 1.9$

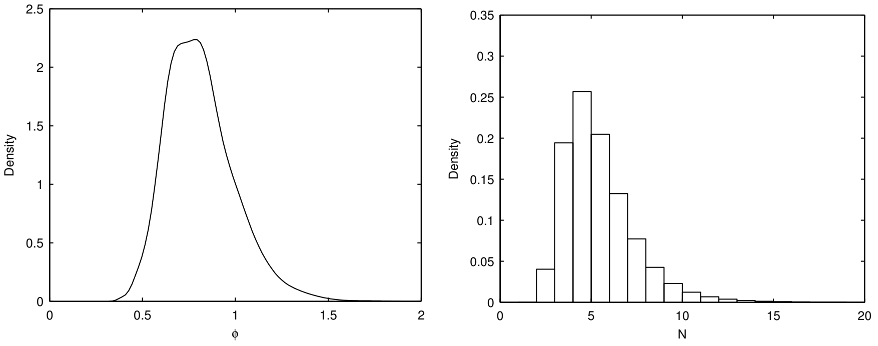

(c) Simulation B_3: $\phi_{\text{est}} = 0.81$, (0.52, 1.22), mode(N_{est}) = 4, mean(N_{est}) = 4.9, $\sigma(N_{\text{est}}) = 2.0$

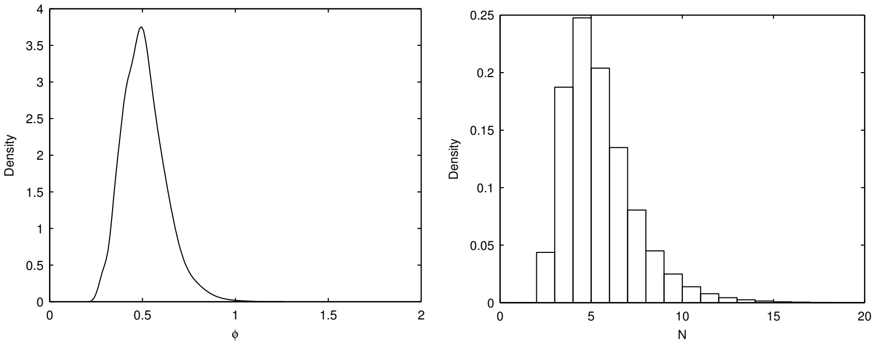

(d) Simulation B_4: $\phi_{\text{est}} = 0.51$, (0.32, 0.78), mode(N_{est}) = 4, mean(N_{est}) = 4.9, $\sigma(N_{\text{est}}) = 2.0$

Figure 9: Simulation B: Fits to ϕ (left) and N (right)

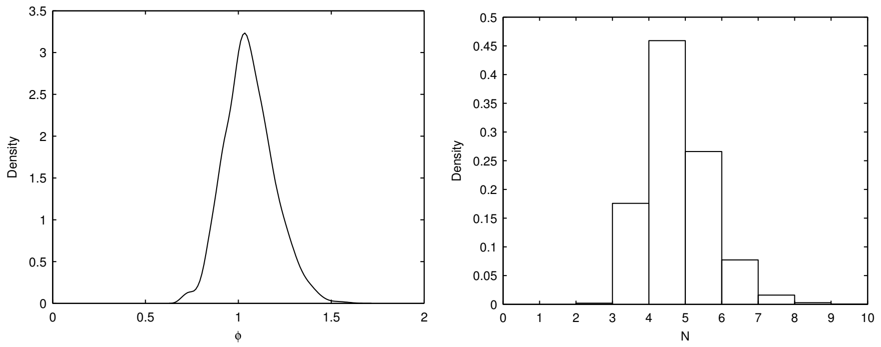

(a) Simulation C_1: $\phi_{\text{est}} = 1.06$, (0.83, 1.34), mode(N_{est}) $= 4$, mean(N_{est}) $= 5.0$, $\sigma(N_{\text{est}}) = 0.94$

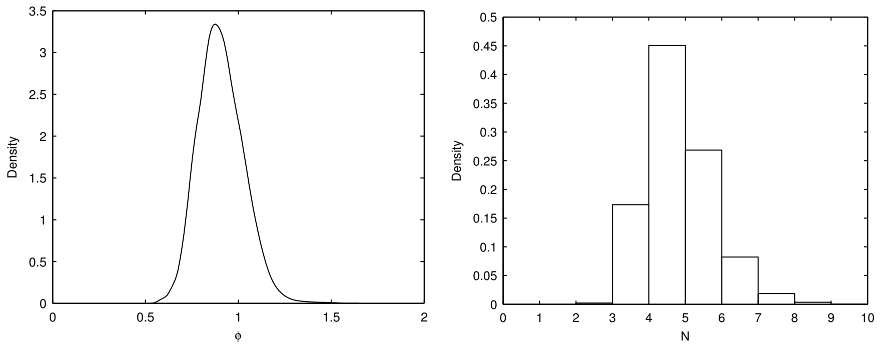

(b) Simulation C_2: $\phi_{\text{est}} = 0.91$, (0.70, 1.15), mode(N_{est}) $= 4$, mean(N_{est}) $= 4.3$, $\sigma(N_{\text{est}}) = 0.96$

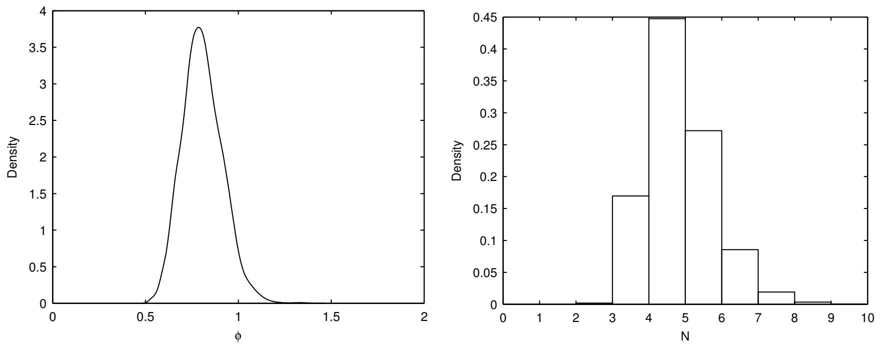

(c) Simulation C_3: $\phi_{\text{est}} = 0.81$, (0.62, 1.03), mode(N_{est}) $= 4$, mean(N_{est}) $= 4.3$, $\sigma(N_{\text{est}}) = 0.96$

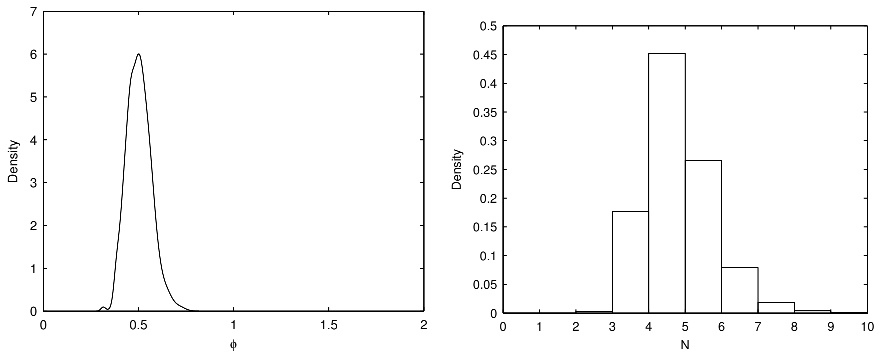

(d) Simulation C_4: $\phi_{\text{est}} = 0.50$, (0.39, 0.65), mode(N_{est}) $= 4$, mean(N_{est}) $= 4.3$, $\sigma(N_{\text{est}}) = 0.97$

Figure 10: Simulation C: Fits to ϕ (left) and N (right)

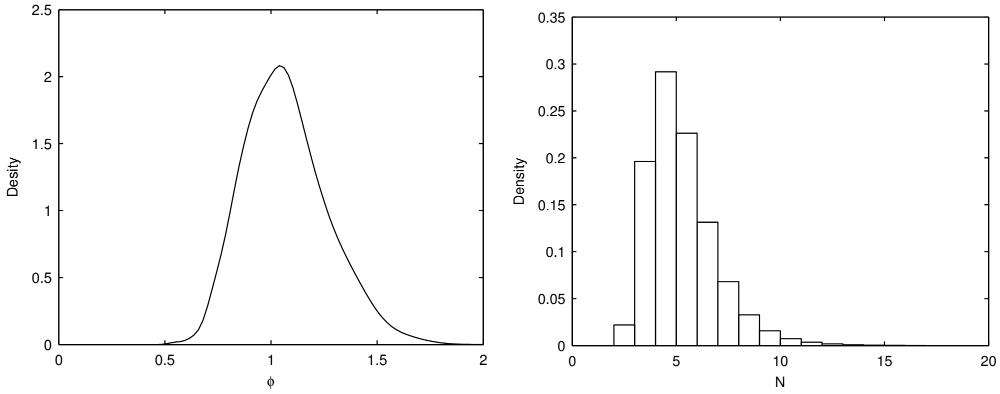

(a) Simulation D_1: $\phi_{\text{est}} = 1.07$, (0.74, 1.47), mode$(N_{\text{est}}) = 4$, mean$(N_{\text{est}}) = 4.8$, $\sigma(N_{\text{est}}) = 1.7$

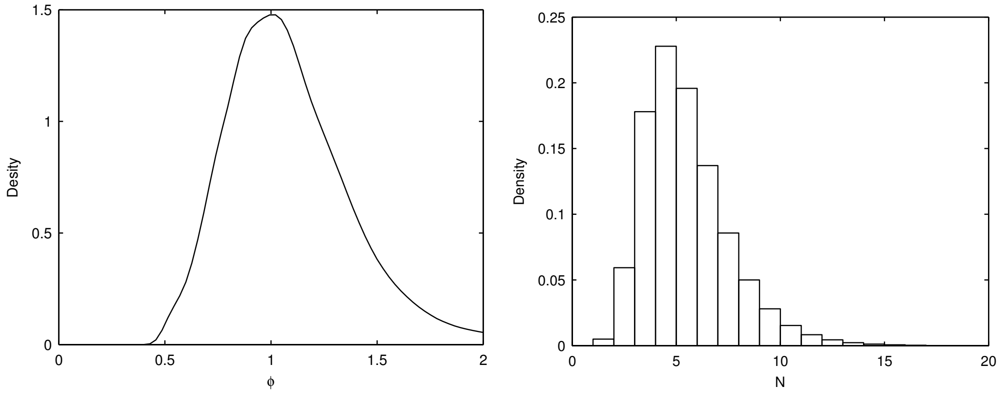

(b) Simulation D_2: $\phi_{\text{est}} = 1.09$, (0.62, 1.85), mode$(N_{\text{est}}) = 4$, mean$(N_{\text{est}}) = 5.0$, $\sigma(N_{\text{est}}) = 2.1$

Figure 11: Simulation D: Fits to ϕ (left) and N (right)

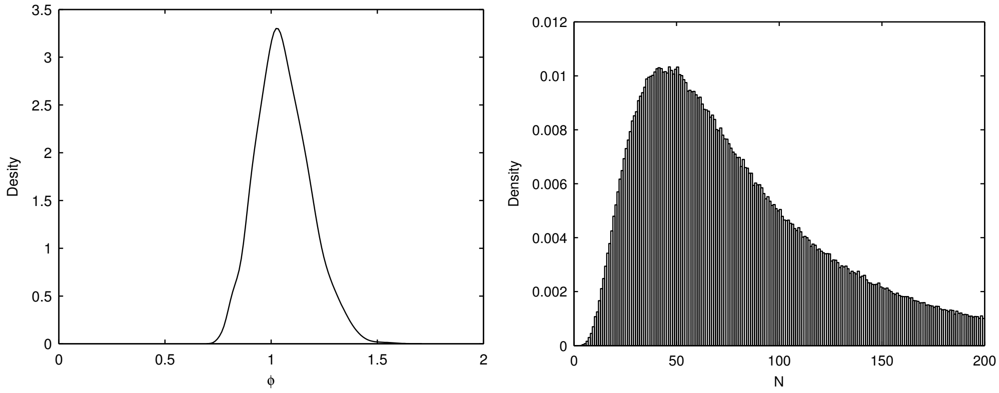

(a) Simulation E_1: $\phi_{\text{est}} = 1.06$, (0.83, 1.33), mode$(N_{\text{est}}) = 46$, mean$(N_{\text{est}}) = 94.4$, $\sigma(N_{\text{est}}) = 79.4$

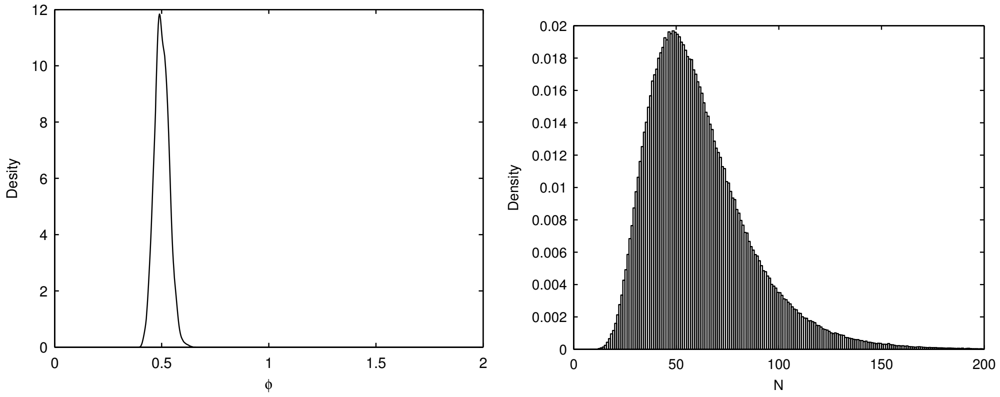

(b) Simulation E_2: $\phi_{\text{est}} = 0.50$, (0.44, 0.57), mode$(N_{\text{est}}) = 48$, mean$(N_{\text{est}}) = 60.8$, $\sigma(N_{\text{est}}) = 25.3$

Figure 12: Simulation E: Fits to ϕ (left) and N (right)
